# Targeting autophagy in pancreatic cancer: The cancer stem cell perspective

**DOI:** 10.3389/fonc.2022.1049436

**Published:** 2022-11-24

**Authors:** Dimitrios Troumpoukis, Adriana Papadimitropoulou, Chrysanthi Charalampous, Paraskevi Kogionou, Kostas Palamaris, Panagiotis Sarantis, Ioannis Serafimidis

**Affiliations:** ^1^ Center of Basic Research, Biomedical Research Foundation of the Academy of Athens, Athens, Greece; ^2^ First Department of Pathology, Medical School, National and Kapodistrian University of Athens, Athens, Greece; ^3^ Molecular Oncology Unit, Department of Biological Chemistry, Medical School, National and Kapodistrian University of Athens, Athens, Greece

**Keywords:** autophagy, pancreas, pancreatic cancer, PDAC - pancreatic ductal adenocarcinoma, Cancer Stem Cell (CSC), pancreatic cancer stem cells, hydroxychloroquine

## Abstract

Pancreatic cancer is currently the seventh leading cause of cancer-related deaths worldwide, with the estimated death toll approaching half a million annually. Pancreatic ductal adenocarcinoma (PDAC) is the most common (>90% of cases) and most aggressive form of pancreatic cancer, with extremely poor prognosis and very low survival rates. PDAC is initiated by genetic alterations, usually in the oncogene KRAS and tumor suppressors CDKN2A, TP53 and SMAD4, which in turn affect a number of downstream signaling pathways that regulate important cellular processes. One of the processes critically altered is autophagy, the mechanism by which cells clear away and recycle impaired or dysfunctional organelles, protein aggregates and other unwanted components, in order to achieve homeostasis. Autophagy plays conflicting roles in PDAC and has been shown to act both as a positive effector, promoting the survival of pancreatic tumor-initiating cells, and as a negative effector, increasing cytotoxicity in uncontrollably expanding cells. Recent findings have highlighted the importance of cancer stem cells in PDAC initiation, progression and metastasis. Pancreatic cancer stem cells (PaCSCs) comprise a small subpopulation of the pancreatic tumor, characterized by cellular plasticity and the ability to self-renew, and autophagy has been recognised as a key process in PaCSC maintenance and function, simultaneously suggesting new strategies to achieve their selective elimination. In this review we evaluate recent literature that links autophagy with PaCSCs and PDAC, focusing our discussion on the therapeutic implications of pharmacologically targeting autophagy in PaCSCs, as a means to treat PDAC.

## Introduction

Pancreatic Ductal Adenocarcinoma (PDAC) is the most common form of pancreatic cancer and is expected to become the second-leading cause of cancer-related deaths worldwide by 2030 (1, 2). PDAC origin remains a controversial issue but most studies support the notion that it arises from the uncontrollable proliferation of the ductal cells of the exocrine compartment, resulting in the development of a highly aggressive neoplasm (3). The molecular profiling of PDAC includes multiple gene expression alterations and copy number aberrations with KRAS (Kirsten rat sarcoma viral oncogene homolog) mutations accounting for more than 90% of the cases. However, further cancer progression requires additional mutations including tumor suppressor protein p53 (TP53), cyclin-dependent kinase inhibitor 2A (CDKN2A) and SMAD family member 4 (SMAD4) (4, 5). Treatment strategies for PDAC include tumor resection in combination with chemo/radio therapies but the efficacy is very limited as most patients relapse and eventually die from metastasis within 5 years (2).

In PDAC, approximately 1% of the total tumor mass consists of Pancreatic Cancer Stem Cells (PaCSCs) that feature auto-renewal and differentiation characteristics that allow them to generate multiple and genetically diverse cancer cell lineages (6). PaCSCs were initially described by Li et al. as cells that uniquely express a combination of CD24/CD44/EpCAM surface markers, and display enriched tumor initiating capacity when transplanted in immunocompromised mice (7). Over the last few years, investigation of the PaCSC population has attracted significant attention and is now being recognised as the main source of tumor heterogeneity and plasticity, and a niche of major importance for pancreatic tumor initiation and progression (8). In addition to CD44, CD24 and EpCAM(ESA), other markers that have been subsequently employed to characterise PaCSCs include CD133, ALDH1, CXCR4 and DCLK1, however, expression of these proteins is not restricted to PaCSCs exclusively (9–12). Many diverse signaling pathways have been found to operate in PaCSCs, including Notch, WNT, Hippo, Sonic-Hedgehog, mTOR and PI3K/Akt. These pathways actively maintain stemness and ensure an increased metastatic potential, but at the same time promote chemoprevention and resistance to conventional therapies (13).

A major cellular process that was found to be critically altered during PDAC initiation and progression is autophagy (14). Autophagy is a highly conserved “self-digestion” process that involves the catabolism of dysfunctional compounds, damaged organelles and engulfed pathogens to maintain cellular integrity and survival. Autophagy is achieved by the enclosure of this cytoplasmic “waste” cargo inside double-membrane vesicles and their concomitant translocation to lysosomes for degradation. Normally, autophagy acts as a major regulator of homeostasis, however under stressful conditions (hypoxia, starvation etc) mainly induced by disease (including cancer), the degradation of subcellular elements is accelerated in order to recycle the macromolecules and render them available to fulfill energy requirements (15–17). Major autophagic signals and regulators include the mammalian target of rapamycin (mTOR), AMP-activated protein kinase (AMPK), WNT and TGFβ (18–20).

In this concise review, we evaluate the literature, outline current evidence on the role of autophagy in PaCSCs, and discuss how this knowledge can lead to more effective therapies against PDAC.

## Autophagy in PDAC – A double-edged sword

Increased autophagic activity in PDAC has long been recognized as a major contributing factor in tumor survival, progression and metastasis. Through autophagy, PDAC tumors gain vital resources for the maintenance of their integrity, and this is achieved *via* mechanisms that have been expertly reviewed in great detail elsewhere (more recently by Gillson et al, 2022) (14). Our intention here is to focus on the seemingly contradictory and often debated dual (positive and negative) role of autophagy in PDAC. In this context, autophagy has been shown to attenuate the development of preneoplastic lesions in early pancreatic carcinogenesis, whereas, at more advanced stages, it was shown to promote tumor development. Disruption of the vital autophagy related genes *atg7* or *atg5* induces the development of benign pancreatic intraepithelial neoplasia (PanIN) in mice harboring the oncogenic Kras^G12D^ mutation. However, despite the fact that spontaneous adenomas do occur, these are not able to progress to a more malignant state (21–23). Autophagy is up-regulated in various PDAC cell lines and is found to be elevated in PanINs as they progress towards PDAC. Consequently, inhibition of autophagy with the use of chroloquine or with shRNA against *atg5* suppresses their growth *in vitro* and attenuates tumor development in pancreatic cancer mouse models (24). This effect was attributed to elevation of ROS (Reactive Oxygen Species) levels, increased DNA damage and impaired mitochondrial function. Resulting tumors retain their benign character and do not evolve to a more malignant state, indicating that autophagy assists tumor initiation at first but then acts as tumor suppressor, blocking progression to more advanced stages. Similar observations were made with the loss of *atg5* or *atg7* in mice harboring oncogenic KRAS mutations. Biallelic loss of *atg5/7* reinforced tumor initiation but repressed the transition of PanINs to PDAC in mice carrying a wild-type *p53* allele. On the contrary, when *p53* is deleted, loss of *atg5/7* results in accelerated tumor progression indicating that *p53* status can determine whether autophagy will act as a tumor suppressor or accelerate tumor development (22, 23). In a later study, Görgülü et al, showed that biallelic deletion of *atg5* in the presence of oncogenic KRAS stimulated acinar-to-ductal metaplasia (ADM), which progressed to PanIN stage 1 but did not eventually lead to PDAC. In contrast, mice carrying just one wild-type copy of the *atg5* gene were able to develop PDAC and exhibited increased invasive capacity with a higher frequency than their wild-type (KRAS : ATG5+/+) counterparts (25).

Interesting results regarding the cumulative role of autophagy in PDAC were also extracted from two mouse models, in which hallmark KRAS^G12D^ mutation was combined with two distinct heterozygous Trp53 loss-of-function mutations, frequently encountered in human pancreatic carcinomas (Trp53R172, Trp53172H) (26, 27). In these models, one Trp53 allele is inactivated *via* a loss-of-function mutation, while the second one is subsequently lost *via* loss of heterozygosity (LOH), at a later stage of tumor progression (28). These models do not bear a complete deletion of Trp53, and therefore emulate human disease pathology more faithfully, thus enabling the investigation of the complex interplay between mutant Trp53 and autophagy regulation. In both models, pancreas-specific autophagy inhibition was achieved *via* a Cre-mediated genetic ablation of ATG7 with conflicting results. While in KRasG12D/+; Trp53R172H/+ animals, ATG7 ablation impeded tumorigenesis, by reducing incidence of both pre-invasive and terminal PDAC lesions (27), KRasG12D/+; Trp53R172/+ mice were characterized by an increased abundance of both acinar-to-ductal metaplasia (ADM) and pancreatic-intraepithelial neoplasia (PanIN) foci as well as more foci of invasive pancreatic tumors (26). The contradictory data derived from the above studies confirm the context-dependent role of autophagy in tumor evolutionary routes, which seems to be largely affected by the genetic background of the tumor-initiating cells.

The dual role of autophagy in PDAC biology has highlighted the importance of deciphering whether this process has a predominantly positive or negative effect in tumor development. Clarification of this important point will determine whether autophagy should be pharmacologically stimulated or inhibited in order to provide an effective treatment option for PDAC, with consensus so far favoring the later hypothesis.

## Autophagy in pancreatic cancer stem cells

**Figure 1:**
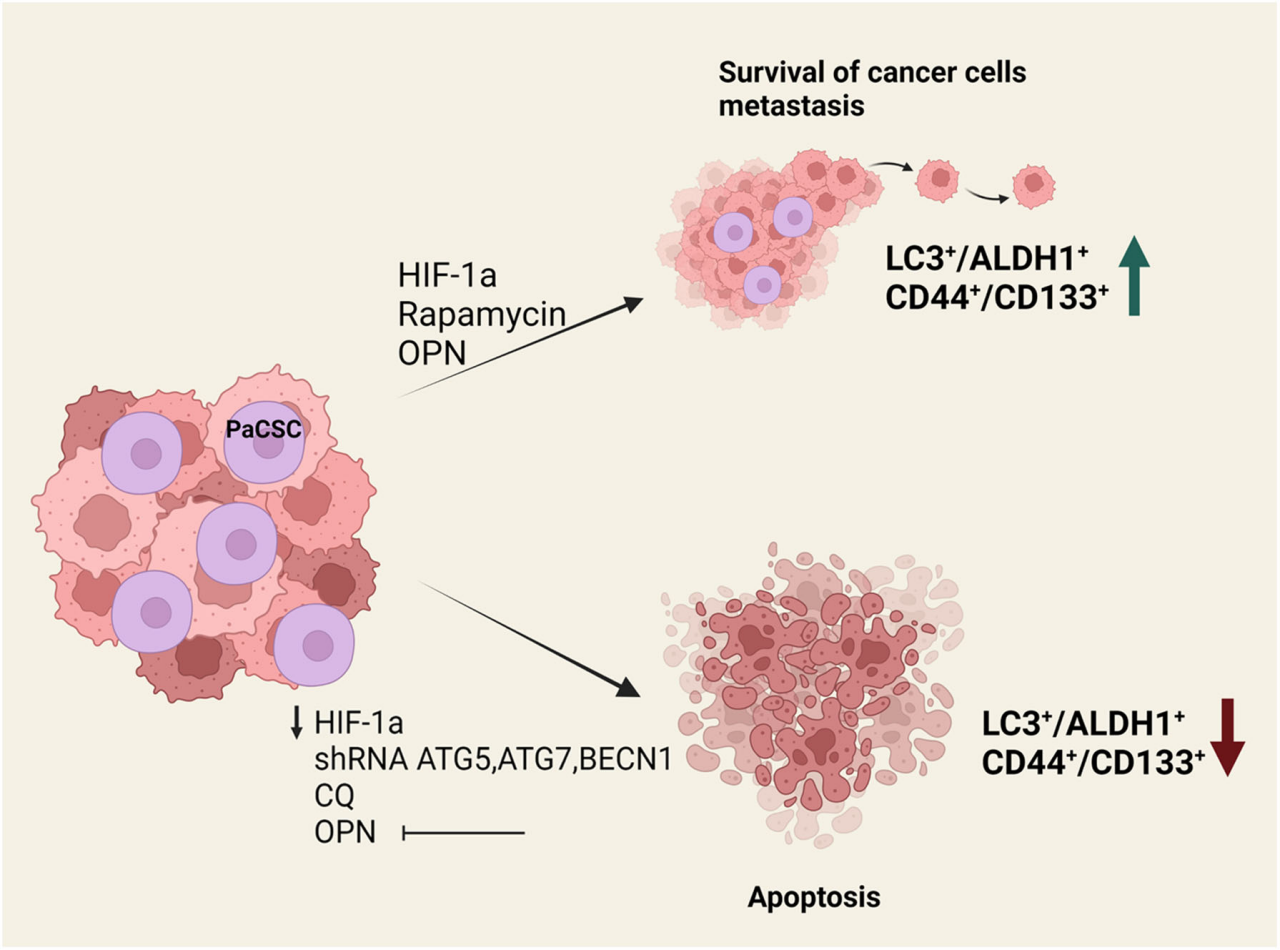
Effects of autophagy perturbation in PaCSCs maintenance and function. *In vitro* and *in vivo* experiments have shown that the presence of HIF-1a, rapamycin or osteopontin increase the survival of cancer stem cells and help metastasis. This is associated with an increase in LC3+/ALDH1+ and CD44+/CD133+ surface marker expression. On the contrary, when there is a reduction of HIF-1a or osteopontin, when *atg5*, *atg7* and *becn1* are silenced, or in the presence of chloroquine, cancer stem cells undergo apoptosis.

Cancer stem cells have been shown to rely heavily on autophagic processes for the maintenance of their stemness, their survival under hypoxic and other stress conditions and the development of resistance to therapies (29). The study of autophagy in PaCSCs has been attracting increasing attention over the last decade due to the realization of its crucial involvement in PDAC development and its potential role in PDAC therapies (**Figure 1**). Rausch et al. was the first to demonstrate an interaction between PaCSCs, hypoxia and autophagy, initially by studying fixed patient-derived PDAC samples where he revealed strong co-expression of markers for CSCs (CD44, CD24), hypoxia (CA-IX) and autophagy (BECLIN1, LC3) (30). During hypoxia, cells from the established cell line MIA-PaCa2 that features high CSC properties are able to survive and migrate, whereas BxPc-3 cells with limited CSC properties cannot respond efficiently and eventually undergo apoptosis (30). The higher stemness of MIA-PaCa2 cells is due to several key mutations that they carry. These include amino-acid substitutions in KRAS and TP53 (G12D and R248W, respectively) and a complete loss of CDKN2A (31, 32). On the contrary, BxPc-3 cells appear to have physiological KRAS while they carry a different amino-acid substitution in TP53 (Y220C) and display complete loss of both CDKN2A and SMAD4 proteins. These genotypic differences are considered to be responsible for the higher stemness of MIA-PaCa2 cells, indirectly leading to the suppression of non-canonical Wnt signaling (33). In this context, autophagosomes and expression of autophagy-related genes (beclin1, atg3, atg4b, and atg12) were found to be significantly elevated in CSCs of the MIA-PaCa2 cell-line but not in BxPc-3 cells. Similarly, Zhu et al. has shown that in the stressful environment of hypoxia, pancreatic cancer cells with stem cell-like properties (isolated by means of CD133 expression) displayed a significant degree of metastatic potential, self-renewal ability and elevated expression of the autophagy-related proteins LC3-II and BECLIN1 (34). Moreover, HIF-1a has been positively correlated with autophagy (35) as migrating cells with stem cell features exhibited concurrent up-regulation of HIF-1a and autophagy-related genes (ATGs). This correlation was further confirmed by evidence that HIF-1a down-regulation by RNA silencing results in reduced autophagy (34). Co-expression of the autophagy marker LC3 with stem cell markers CD133, CD44 and ALDH1 in pancreatic cancer tissue samples is another example in support of the positive correlation between autophagy and cancer cell stemness (36). Interestingly, the same study showed that pancreatic cancer patients with increased co-expression of LC3 and ALDH1 correlated with poor Progression Free Survival (PFS) and worse Overall Survival (OS). PANC-1 cells transfected with lentivirus carrying shRNA against *atg5*, atg7 and *becn1* exhibited a significant decrease in CD44, CD133 and ALDH1 expression. Reduced levels of expression of these CSC markers concomitantly affected the stem cell properties of the transfected cells. Their self-renewal capacity and their proliferation potential were reduced, as these cells formed less spheres and exhibited impaired growth in culture compared to their control counterparts (36). Furthermore, when the transfected cells were transplanted into NOD/SCID mice, tumor volume was consistently lower compared to control mice and displayed resistance to gemcitabine. These results were further confirmed by pharmacological regulation of autophagy with the addition of the autophagy inhibitor chloroquine (CQ) and the inducer rapamycin. Levels of LC3/ALDH1 and CD44/CD133 expression were reduced by CQ treatment but showed significant increase in the presence of rapamycin (36). Additionally, osteopontin (OPN), which was previously reported to positively regulate sphere forming ability of cancer cells, was found to increase the levels of CD44, CD133, ALDH1 and LC3-II in various pancreatic cell lines, an effect that can be reversed by attenuating osteopontin activity or blocking autophagy altogether. After suppression of the OPN-mediated signaling pathways, it was revealed that NF-kB is a major contributor of OPN-stimulated autophagy and CSC activity. This observation is in agreement with other studies that have identified NF-kB as an inducer of autophagy under stressful environmental conditions such as heat shock (37). Finally, recent studies by Qin et al. showed that the stem cell-like properties of pancreatic cancer cells were reduced after the inhibition of the Nutrient-deprivation Autophagy Factor-1 (NAF-1), an autophagy-related protein localized in the outer mitochondrial membrane that is mainly involved in the maintenance of mitochondrial integrity (38–41). Pancreatic CSCs display an increased sensitivity to defects in mitochondrial homeostasis due to their predominant dependence on oxidative phosphorylation (OXPHOS) for their survival (42). NAF-1 inhibition therefore significantly affects stem cell properties of PaCSCs and mitigates their invasive capacity (41).

## Targeting autophagy in PDAC

The majority of patients with PDAC are diagnosed in advanced stages of the disease, when the tumor is inoperable and surgery is no longer a viable treatment option. Chemotherapy and radiotherapy are first-line palliative treatments for PDAC patients and the standard chemotherapeutic drug commonly used was Gemcitabine until Folfirinox (a combination of 5-fluorouracil, folinic acid, irinotecan and oxaliplatin) proved to be more efficient in increasing OS (43).

Targeting autophagy in combination with chemotherapy has emerged as an attractive and very promising strategy in confronting PDAC. A variety of autophagy inhibitors, with distinct substrate specificities, have been developed and evaluated *in vitro* on cancer cell-lines, as well as *in vivo* on mouse models of PDAC. Based on their target molecules, the existing inhibitors can be segregated into two main classes: those that aim at constraining the initial step of autophagosome formation (44, 45) and those that block the merging of autophagosomes with lysosomes by altering the acidic environment of lysosomes (46, 47). The first class encompasses agents that impede the generation of the two core autophagy initiation multi-protein complexes, ULK1 and PI3KC3-C1 (48, 49). The main representatives of this class are MRT68921 (50, 51), SBI-0206965 (52–54) and SBI-7455 (55), which selectively target ULK1 kinase, and Spautin-1 (56, 57) and SAR405 (58, 59), which selectively suppress the PI3KC3-C1 complex. To this day, none of these compounds have progressed further into clinical studies on human patients.

On the contrary, the compound Hydroxychloroquine (HCQ) -a derivative of chloroquine and the major representative of the second class of autophagy inhibitors (60) - has been assessed in a number of clinical trials for PDAC, either as a monotherapy, or in combination with standard chemotherapy regimens or novel classes of anti-proliferative drugs (**Table 1**). Initially, a clinical trial investigated the effect of monotherapy using daily administrations of Hydroxychloroquine, however the drug yielded no significant responses (61). On the contrary, two phase 1/phase 1b/2 clinical trials that used a combination of gemcitabine and HCQ, provided encouraging data as there was reduction of the CA19.9 tumor marker, an increase in LC3-II staining and an improved PFS. Despite these very promising observations however, no safe conclusions could be drawn by these two studies due to the small number of patients and the lack of randomization (62, 63). More recently, a randomized phase II clinical trial was conducted using Gemcitabine together with nab-paclitaxel, with or without HCQ, in patients with advanced pancreatic cancer. Although the OS or PFS were not significantly improved, the study showed that the response rate was improved in HCQ-treated patients, therefore it was proposed that HCQ be included at the preoperative stage (61, 64).

**Table 1:** Clinical trials for PDAC that include targeting of autophagy.

CLINICAL TRIAL	REGIMEN	PHASE	RESULTS	REFERENCE
NCT01273805	Hydroxychloroquine (HCQ)	Phase II	No significant responses	Wolpin et al. 2014 (61)
NCT01128296	HCQ with Gemcitabine	Phase I/II	Decrease in CA19-9 Surgical oncologic outcomes were encouraging	Boone et al. 2015 (62)
NCT01777477	HCQ with Gemcitabine	Phase I	No dose-limiting toxicities Median time to progression was 4 months Median overall survival was 7.6 months	Samaras et al., 2017 (63)
NCT01506973	Gemcitabine hydrochloride and nab-paclitaxel (GA) ± HCQ	Phase II	Overall survival at 12 months was 41% in the HCQ group and 49% in the non-HCQ group Overall response rate was 38.2% in the HCQ group and 21.1% in the non-HCQ group	Karasic et al., 2019 (64)

Many attempts have been made in order to combine autophagy inhibition with novel cancer immunotherapy protocols that are currently under development. Yamamoto et al. showed that autophagy inhibition increases expression of MHC-I molecules on the surface of PDAC cells, leading to increased infiltration of immune cells, thus demonstrating that autophagy inhibition combined with immunotherapy could be a promising therapeutic option against PDAC (65). Nevertheless, the sole study to this day performed on pancreatic cancer patients treated with a combination of HCQ, gemcitabine, nab-paclitaxel and avelumab (an anti-PD-L1 antibody) was terminated due to undesirable side effects. Further clinical trials that combine HCQ with MAPK inhibitors for advanced and metastatic pancreatic cancer are currently under way (66).

In addition to the synthetic agents mentioned above, a number of natural derivatives have demonstrated a capacity to interact in multiple ways with the autophagic pathways, and can potentially be used in PDAC treatment. Even though their exact mechanism of action is unclear, they seem to converge to a common path of apoptosis inhibition. Fudan-Yueyang-Ganoderma lucidum (FYGL; a proteoglycan extracted from Ganoderma lucidum) and Alantolactone demonstrated inhibitory effect in late-stage of autophagy along with increased apoptotic potential, mediated by elevated ROS production and suppression of STAT3 and Bcl-2 respectively (67–70). Curcumin is also capable of inducing pro-apoptotic effects, by increasing the BAX/Bcl-2 ratio (71), while ursolic acid (UA) downregulated autophagy, predominantly *via* inducing cell cycle arrest (72).

## Discussion – prospects of targeting autophagy in PaCSCs

The destruction of CSC niches is essential in order to achieve PDAC therapy and avoid cancer recurrence, however CSC niches in PDAC tumors are protected by the low-oxygen tumor environment and this is a major contributing factor to anti-cancer therapy resistance (29). Possible treatments could arise from the targeting of autophagy in PaCSCs and several preclinical studies are exploring the possibility of eliminating PaCSC niches by perturbing autophagy. Signaling pathways that are active in PaCSCs, such as NOTCH, WNT and SHH, are being explored as possible targets for impairing pluripotency in combination with autophagy targeting (9). On the other hand, the fact that PaCSCs reside in the anatomically distinct regions of a niche, which is often pharmacologically inaccessible, necessitates the exploration of alterative properties of PaCSCs as targets for therapy. Cancer dormancy is characterized by attenuation of cell proliferation and the transition to a quiescence-like state. It has been shown that dormancy contributes significantly to cancer relapse and induction of metastasis. Conventional anticancer therapies usually target proliferating cells hence the acquisition of a dormant phenotype results to the evasion of treatments and worse overall survival. The biological mechanisms and signaling pathways regulating the dormant phenotype of tumor cells also apply to CSC behavior, and recent studies indicate that autophagy plays a vital role in the entrance of CSCs to a dormant state, the maintenance of their survival and their reactivation under specific conditions (73).

In addition to the pre-clinical studies, efforts to effectively tackle PDAC currently explore the clinical use of small molecules that target autophagy, in combination with chemotherapy and/or immunotherapy (14). Targeting cancer stemness is currently the basis of ongoing clinical trials on Phase III with paclitaxel/nab paclitaxel and gemcitabine with the addition of napabucasin, a small molecule that inhibits STAT3-mediated gene transcription. The results from phase 1b/2 achieved a Partial Response (PR) of almost 42% and approximate 3% of Complete Response (CR) (74). This enhanced chemotherapy scheme could be complemented with selected compounds that target key components of the autophagic process, in order not only to eliminate tumor-cells but also to eradicate hidden and evading PaCSCs.

Despite the undoubted benefits of autophagy inhibition in cancer treatment, the complex role of this “self-digestion” process in tumorigenesis, coupled with the capacity of tumor stem cells to utilize alternative nutrient sources under starvation conditions, could impose some restrictions to the clinical efficacy of this therapeutic approach. While in the majority of cases autophagy is considered a cytoprotective mechanism that serves as an adaptation system of neoplastic cells in conditions of nutrient scarcity, there is still controversy regarding its overall effects in cancer initiation and progression. Recent experimental evidence, from both *in vitro* and *in vivo* studies, suggests a context-dependent anti-tumor effect of autophagy, mediated by a detrimental effect on cancer stem cell survival and metastasis. Even though autophagy is a process antagonistic to apoptosis, overt autophagic influx can in fact trigger apoptosis under certain conditions by activation of caspase-8 and the diminution of endogenous apoptosis inhibitors (75). Moreover, genetic ablation of ATG5 in a KRAS-driven mouse model of PDAC enhances the metastatic potential of tumor stem cells, implying an anti-metastatic effect of autophagy under certain circumstances, especially during the initial stages of tumor development (25). It has also been shown that neoplastic cells are equipped with an intrinsic ability to circumvent dependency on autophagy and exploit compensatory signal transduction systems, such as Nrf2 signalling and deployment of complementary nutritional sources through the process of micropinocytosis (76). These strategies enable cancer stem cells to survive nutrient stress conditions arising from starvation, thus ensuring their survival and growth. It is therefore implied that potential anti-PDAC pharmacotherapy schemes relying on autophagy targeting in PaCSCs should carefully consider both the tumor-suppressive and the tumor-promoting effects of this action.

## Author contributions

DT, AP and IS wrote the first draft of the manuscript. All authors contributed to the article, reviewed and edited the final manuscript and approved the submitted version.

## Funding

Work in the senior author’s laboratory is supported by funding provided by Boehringer Ingelheim GmbH (CRA: 493341) and the Greek General Secretariat of Research and Innovation (GSRI) (Code: T1EDK-03532). The funders had no role in study design, data collection and analysis, decision to publish, or manuscript preparation.

## Acknowledgments

The authors thank all members of the Stem Cell and Cancer Biology laboratory at BRFAA for critical discussions over the manuscript and wish to apologise to all investigators whose work has not been cited here due to space restrictions.

## Conflict of interest

The authors declare that the research was conducted in the absence of any commercial or financial relationships that could be construed as potential conflict of interest.

## Publisher’s note

All claims expressed in this article are solely those of the authors and do not necessarily represent those of their affiliated organizations, or those of the publisher, the editors and the reviewers. Any product that may be evaluated in this article, or claim that may be made by its manufacturer, is not guaranteed or endorsed by the publisher.
